# Medical Items and News of Interest to the Physician

**Published:** 1871-04

**Authors:** 


### Of Interest to the Profession.

CULLED FROM THE STANDARD MEDICAL JOURNALS OF THE WORLD.

NOTE.—These formulæ are intended only for the regular practitioner of medicine, and should be employed only by him, or under his immediate supervision.

**To Disguise Quinine.**

Give it in strong, hot chocolate.

**Chloral Hydrate.**

The nausea produced by the administration of this remedy can be prevented by giving it in diluted lemon juice.

**Obstinate Hiccough.**

Dr. Jauritz, (*Siglo Midico*), relates a case of a physician who being seized with a serious hiccough, while convalescing from gastric fever, drank in mistake an infusion of mustard, and obtained relief.

**Double-Headed Child.**

Mrs. Findley, the wife of a respectable well-to-do farmer, of Morrow County, Ohio, (*Cin. Med. Repos.*) gave birth to a child with two heads, in October last. Each child has a separate stomach, but bowels in common.

**Delirium Tremens.**

Wm. S. Bowen, M. D., U. S. N., (*Boston Med. & Surg. Jour.*), recommends the use of this drug, and publishes a case where he administered from 20 to 60 grains, in water flavored with ess. of peppermint, in which a rapid recovery was made.

**Chorea.**

Hydrate of chloral is highly extolled, in cases of chorea, by a prominent western practitioner and others. Whether it is permanently beneficial or not, it acts like magic in controlling the unruly muscles, and in allaying the alarm of friends.—*Medical Record.*

**Odontalgia.**

Dr. H. T. Reynolds writes to the *Phila. Med. News* that, "for eighteen months I have been using acetate of lead for tooth ache. I find it to act better than any of the numerous remedies proposed in the books, and in cases where it is applicable the relief is instantaneous. Let the patient apply one or two grains to the cavity and then spit it out. It fails in fewer cases than any remedy I have tried.

**Ozæna.**

Trosseau's favorite injections in ozæna were: No. 1. chlorate of potash, one part; water, one hundred parts. No. 2. nitrate of silver, five parts; distilled water, one hundred parts. No. 3. sulphate of copper or zinc, five parts; distilled water, one hundred parts.—*Lectures on Clin. Medicine.*

**Obstinate Eczema.**

Dr. Chas. R. Drysdale, of London, (*Jour. Cutan. Med.*), places great reliance in a strong solution of caustic potash, in many cases of obstinate eczema of the ears and lower extremities—one of caustic potash to two of water. It is a painful application, but the pain ceases on the application of wet sago to the parts.

**Puerperal Fever.**

Prof. Tyler Smith, in the *American Jour. of Med. Sciences*, says he has treated and cured a desperate case of puerperal fever by injecting ammonia into the veins. He employed about seven minims, diluted with three times that quantity of water. Under this treatment the symptoms of uræmic poisoning disappeared.

**Leucorrhœa.**

Dr. A. H. Kinnear, Metamora, Ill., (*Chic. Med. Jour.*), has given, with success, in twelve marked cases of vaginal or uterine leucorrhœa, none of which were of less than six month's standing, bromide of potassium. The majority of the cases yielded to the treatment in four weeks. He has observed two effects from the use of this drug: the alterative and nervo-sedative.

**Dysentery.**

The physicians of the Montreal, Canada. General Hospital, publish cases of acute dysentery successfully treated with large doses of ipecacuanha. In these cases strict injunctions were given to use the least possible amount of fluid following the admittance into the hospital. The ipecacuanha was given three times daily in powder, in doses from ten to twenty grains.

**Prevention of Scarlatina.**

Dr. Wm. Budd has published a pamphlet on *Scarlet Fever and its Prevention*, in which the author declares that so remarkable has been his success with the method of isolation and disinfection which he has practiced, that for a period of 20 years, he has never known this disease in a single instance, beyond the sick room.

**Biliary Calculi.**

A physician, writing to the *Chicago Med. Jour.*, recommends the use of olive oil, in large doses, for the expulsion of biliary calculi. He gave a pint of oil to one of his patients, and at the expiration of twenty-four hours, more than one hundred biliary calculi passed per anum—more than sufficient in bulk to fill a half pint measure.

---

**Insomnia.**

℞.

Potassii bromidi, ℥ ss;

Aquæ cinnamomi, f. ℥ ij. M.

Sig:—Dessertspoonful a quarter of an hour before the last meal, and the same dose, or three teaspoonfuls repeated at bed-time, for adults. *Dr. Brown-Sequard in Naphey's Ther.*

---

**Anæsthetic Mixture.**

Dr. H. F. Lyster, of Detroit, (*Mich. Unv. Med. Jour.*), uses the following anæsthetic mixture in his practice:—

℞.

Alcoholi, ℥ ss;

Chloroformi, ℥ j;

Æther; Sulphurici, ℥ ij.

Misce. Before administering this anæsthesia, he is in the habit of giving a couple teaspoonfuls of brandy.

---

**Irritability of Stomach.**

Dr. Jno. Sensemen, of Getysburg, Ohio, writes to the Phila *Med. & Surg. Reporter*, that he has had great success with subnitrate of bismuth in irritability of the stomach accompanied with vomiting. He gives an adult from four to eight grains every hour until the vomiting ceases. He believes that in the irritability of dyspepsia it excels all other remedies. Given in from ten to thirty grain doses every three hours, it produces almost specific effects.

---

**Infantile Capilary Bronchitis.**

Dr. Bedford Brown, of Alexandria, Va., reports 21 cases in the following manner: One of the leading objects was to remove mechanical obstruction from mucus exudation in the air tubes. To accomplish this, emetics of ipecacuanha were used in every case but one. When used freely in the primary or dry stage, the effect in a great measure was to prevent the occurrence of the subcrepitant râle. The sulphate of quinia, in combination with ipecac., was used in about two-thirds of the cases treated. The febrile action yielded more rapidly with the quinia than without it. Twenty of his cases recovered.

**Nervous Headache.**

Prof. Wm. A. Hammond, of New York, prescribes as follows:—

℞.

Extracti nucis vomicæ gr. v.

Ferri redacti, ℈ j;

Quinæ sulphatis, gr. x;

Syrupi, q. s. M.

Divide into x x pills.

Sig:—One to be taken three times a day, after meals.

---

**Hysterical Headache.**

When it is accompanied with habitual constipation, and debility, Thomas Hawkes Tanner, M. D., F. L. S., etc., London, prescribes as follows:

℞.

Zinci phosphatis, ℈ j—ij;

Acidi phosphorici dil., f. ℥ iss;

Tincturæ cinchoniæ, f ℥ vj;

Aquæ menth. pip., q. s. ad f. ℥ iij. M.

Sig:—Tablespoonful in half a wine glass of water three times daily.

---

**Indolent Ulcers.**

Starch, glycerine, and carbolic acid is an excellent preparation for healing ulcers and for promoting healthy granulations on wounded surfaces. It is made by heating the glycerine to a boiling temperature, then adding the starch in fine powder, slowly stirring the mixture for a few minutes or until it is brought to the consistency of a soft paste. Then allow it to cool, after which add a small quantity of carbolic acid, and mix thoroughly.—*North-Western Med. & Surg. Jour.*

---

**Cholera Morbus.**

Dr. H. E. Whitehead having had occasion to treat several cases of severe attacks of cholera morbus, and having failed to relieve the first case with opium, ordered for the patient, pills containing one-third to one-half a grain of the extract of belladonna; one to be taken every four hours. The first pill relieved the sense of constriction or knotty pain in the region of the umbilicus, and after the third pill, the bowels were freely moved, although several purgative draughts had been taken previously without effect. After having two grains of the extract, the patient rapidly recovered, with no constipation, headache, or unpleasant symptoms. The result in three other cases with the belladona treatment was equally successful.—*Pacific Med. & Surg. Journal.*

**Functional Indigestion.**

In those forms of indigestion marked by excessive acidity and "heart burn," Dr. Wm. Aitken, recommends the following:

℞.

Sodæ bicarbonatis, gr. xv;

Potassæ, nitratis, gr. iij. M.

For one powder, to be taken two or three times daily.

---

**Habitual Constipation.**

The following is a very excellent pill for this difficulty. It is recommended by Dr. J. M. DaCosta, of Phil.:

℞.

Podophyllin,

Ext. Belladonnæ, āā gr j;

Capsici, gr. v;

Pulv. rhei, gr. xx;

Ft. pil. xx div..

Sig:—Take one pill before every meal.

---

**Hæmorrhoids.**

In recent cases of hæmorrhoids, Prof. Nathan R. Smith, of Baltimore, advocates this formula:

℞

Sulphur lot., ℥j;

Fol. sennæ pulv., ʒj;

Ol. fœnic., gtt. xx.

M. ut f. pulv. Sig: Give a heaping teaspoonful every other night. The parts must be bathed three or four times a day, in cold water, and especially after a stool.—*Med. Record.*

---

**Pneumonitis.**

A. Patton, M. D., of Vincennes, Ind., has treated successfully, during the last five years, 96 cases of pneumonitis, by the exclusive use of carbonate of ammonia, except in the congestive stage, when he sometimes gives 60 drops of chloroform to aid reaction.

He says that if the remedy be given early in the disease, in from 5 to 10 grain doses, regularly every two hours, night and day, it will, in almost every case, so limit the exudation as not only to greatly lessen the amount of hepatization, but to insure a prompt and rapid absorption of the exudation, and occasionally it will entirely prevent hepatization, and terminate the inflammation by resolution. He also alludes to 213 cases treated in this manner by reliable practitioners in Indiana and Illinois, altogether 309 cases, all severe and well marked cases of pneumonitis. In these 309 cases reported, there were 8 deaths, or one in 38.

**Bromide of Potassium for Children.**

E. Montord Martin, of Beaujon Hospital, France, shows the value of this drug in all cases of dentition, where there are aggravating symptoms, and it is improper to give opium. In sleeplessness of infants, in cough merely spasmodic, he gives it in three-fourth grain doses once or twice a day to children from three months to a year old. In purely nervous cough it is equally indicated; but it should *not* be given in diarrhœa, as it increases the difficulty.—*Med. Record.*

---

**Cancrum Oris.**

There is no patient the physician is more anxious about than the one suffering with *gangrene of the mouth.* Dr. W. S. Edgar, of St. Louis, Mo., invites attention, through the *St. Louis Med. & Surg. Journal*, to the prophylactic treatment as all important. Among the ferruginous preparations, the liq. ferri iodidi has his preference in these cases, both for children or adults; it should be given alternately with the citrate of iron and quinia, particularly in malarial regions. The local treatment is no less important; thorough clensing of the ulcers and mouth twice a day with the nebulizer, using a weak solution of the chloroide of sodium or chlorate of potassa; also the permanganate of potassa to correct the offensive odor of the breath, and stimulate healthy granulation.

If any eschorotic is indicated, a strong solution of sul. copper—xxx gr. to the ounce of water—or paste of permang. of pot.

---

**Cancer of the Liver.**

Dr. S. N. Davis of Chicago, Ill., (*Chic. Med. Exam.*), in an interesting clinical lecture on this subject, gives the following treatment:

"Pay particular attention to the diet. Milk and farinaceous articles are the best. Meat should be used only sparingly. For an internal remedy, he has realized the most benefit from some of the arsenical preparations with conium. The following is a good formula:

℞
Arsenici Iodi., gr. jv;
Ex. conium, ʒjss.

Make a mass and divide it into sixty pills.

Sig: Take one, three or four times a day. Anodynes sufficient to relieve pain should be given, selecting those that do not constipate, if possible. Among the best are fluid extract of hops and lettuce. By such treatment the physician can do much to palliate the sufferings of the patient, and delay somewhat the fatal result.

**Syphilis and Scrofula.**

Dr. Maury, surgeon to the Phila. Hospital, is reported to have said in a recent lecture, (*Med. & Surg. Reporter*), that the parallelism between congenital syphilis and scrofula, when closely traced, so approximates that no human being can discriminate between them. He also believes that the unfolding of the next twenty-five years will establish the identity between so-called scrofula and inherited or quarternary syphilis, and this pathway leads him to the broad field of legalized prostitution, of which he is an advocate.

---

**Sea Sickness.**

Dr. O. Rapin, of Switzerland, says that he has found that the nausea and the vomiting produced by swinging and sea-sickness, can be arrested by applying to the epigastrium a layer of wadding dipped in collodion. It should extend from the xiphoid cartilage to the umbilicus, and be left until it falls off. If the adhesion should be imperfect, the application should be renewed. Several persons have tried this plan with benefit. The explanation given is, that the action of the peripheral nerves is interrupted just in the same way as the pain of calculi in the bile-passage or ureters is sometimes mitigated by the application of castor oil and collodian.—*British Med. Journal.*

---

**Obstruction of the Intestines.**

Dr. M. M. Brown, of Ithaca, recommends, in the *Med. & Surg. Reporter*, the injection of Seidlitz powders, in *invagination* and *intussusception* of the Intestines. He gives the details of a case in which the usual remedies failed to remove the obstruction and bring about a movement of the bowels, when he resorted to the original and novel plan of dissolving a blue powder in a pint of warm water and injecting it into the bowels, following it immediately by the dissolved white powder. The result was immediate relief from pain, a free discharge of the bowels, and removal of the obstruction.

---

**Chronic Pleurisy.**

Wm. Aitken, M. D., of Edinburgh, is in the habit of prescribing as follows in chronic pleurisy:

℞.

Pulveris digitalis,

Pulveris scillæ,

Pilulæ hydrargyri, āā, gr. jss. M.

For one pill, two or three times a day, as a diuretic. He also recommends the following lotion to be applied to the chest by means of lint saturated with it, and oiled-silk over all:—

℞.

Hydrargyri chlo. corrssivi, gr. jv;

Tinct. iodini compositæ, f. ʒ jv;

Glycerinæ, f. ℥ iij;

Aqua distillatæ, f. ℥ jvss. M.

---

**Chorea.**

The subjoined formulæ are recommended by Dr. T. Curtis Smith, Middleport, Ohio, in the *Cincinnati Lancet & Observer*, in chorea, in conjunction with strong salt baths, attended with much friction:

℞

Tr. actea racemoræ,

Tr. hyoscyami,

Syrp. simplic., āā f. ℥ ij. M.

Sig: Tablespoonful every six hours; also

℞

Ferri sulphas, ℈ iij;

Zinci sulphas, ℈ jss;

Quiniæ sulphas, ℈ j.

M. ft. pil. xx. Sig: One three times daily.

---

**Asthma.**

Dr. Jas. S. Bailey, of Albany, says:—Having during the last fifteen years, prescribed many of the remedies mentioned, with indifferent and uncertain success, after much careful study, I have prepared the following formulary, which has, during more than half this period, acted to my entire satisfaction. I now place it at the disposal of the profession, hoping it may meet with the same success in their hands as mine, in relieving the distress of this unfortunate class of sufferers. It is as follows:

℞
Syrup of tar compound, ℥ iv;
Sulphuric ether, ℥ ij;
Pulv. gum acacia, ʒ vj. M.

Take one teaspoonful every two or three hours until relieved. Oftentimes two or three doses are quite sufficient to relieve an aggravated attack of spasmodic asthma. As the syrup of tar compound is my own preparation and not pharmaceutical, it is necessary to give the formulary for its preparation, which is as follows:

℞

Picis liquidæ, ℥ iv;

Scillæ acet, O. j;

Antim. et potass. tart., gr. xvj;

Magnesiæ carb., ℥ ss;

Sacch. albi., ℥ xxx;

Sul. ether;

Spts. vini rectificati, āā f. ℥ j.

Mix the tar and carb. magnesia in a mortar, adding the alcohol first and then the ether, then add the acet. scillæ and throw the whole upon a stout filter. Having added sufficient acet. scillæ (if may be,) to make the filtered liquid measure O. j., proceed to make the syrup by the usual U. S. P. formulary, taking care to apply a gentle heat. Lastly, strain through flannel and add the tart. antimonii in solution.

After the paroxysm is relieved, the patient is left in a relaxed and debilitated condition; it is then necessary to resort to a tonic course of treatment. A generous diet, with simple bitters or some mild preparation of iron, is generally sufficient to restore the system to its accustomed health.—*Record.*

### HOW TO MAKE FARM LIFE ATTRACTIVE.

1. By less hard work. Farmers often undertake more than they can do well, and consequently work too early and too late.

2. By more system. The farmers should have a time to begin and stop labor. They should put more mind and machinery into their work. They should theorize as well as practice, and let both go together. Farming is healthy, moral, and respectable; and, in the long run, may be made profitable. The farmer should keep good stock and out of debt.

3. By taking care of health. Farmers have a healthy variety of exercise, but too often neglect cleanliness, eat irregularly and hurriedly, sleep in ill-ventilated apartments, and expose themselves needlessly to cold.

4. By adorning the home. Books, papers, pictures, music and reading should all be brought to bear upon the in-door family entertainments; and neatness and comfort, order, shrubbery, flowers and fruits should harmonize all without. There would be fewer desertions of old homesteads if pains were taken to make them agreeable. Ease, order, health, and beauty are compatible with farm life, and were ordained to go with it.—*Boston Jour. Chemistry.*

LIBERAL.—Fougara, the French druggist of New York, has given $1,000, to the fund for the relief of wounded of the French Army. He wants to be one of a hundred more to raise a hundred thousand dollars for the same purpose.

MAIMED SOLDIERS.—The books in the United States Pension Office show that of those who are drawing pensions, 5,006 have lost one arm, 4,627 one leg, 350 both arms, 42 both legs, and 21 an arm and a leg.

